# Cell type‐specific molecular profiling of the postmortem human hippocampus in the early stages of Alzheimer's disease and Huntington's disease

**DOI:** 10.1002/alz70855_100462

**Published:** 2025-12-23

**Authors:** Ester Siantoputri

**Affiliations:** ^1^ Rockefeller University, New York, NY, USA

## Abstract

**Background:**

The hippocampus, a critical region for learning, memory, and spatial cognition, is affected early in Alzheimer's Disease (AD), while it remains resilient in Huntington's Disease (HD).

**Method:**

We utilized Fluorescent Activated Nuclei Sorting (FANS) followed by bulk RNA and ATAC sequencing to characterize major neuronal and glial cell types from postmortem human hippocampus across AD Braak stages 0‐IV and HD.

**Result:**

While there were minimal alterations to the hippocampus in HD, we observed varying numbers of significant differentially expressed genes in different hippocampal cell types in AD Braak I‐II and Braak III‐IV. Most differentially expressed genes were observed in the inhibitory neurons.

**Conclusion:**

Transcriptomic changes in the hippocampus precede AD neuropathology and clinical diagnosis, and are highly disease‐ and cell type‐ specific.

